# Repairing left ventricular outflow after aortic composite graft pseudoaneurysm: case report

**DOI:** 10.1186/s43044-022-00307-4

**Published:** 2022-11-08

**Authors:** Rita Caldeira da Rocha, Kisa Congo, Manuel Trinca, Álvaro Laranjeira Santos

**Affiliations:** 1 Cardiology Department, Hospital Espírito Santo, Largo Senhor da Pobreza, 7000-811 Évora, Portugal; 2 Departamento Cérebro-Cardiovascular, Évora, Portugal

**Keywords:** Composite graft dehiscence, Bentall redo procedure, Left ventricular outflow reconstruction, Rheumatoid arthritis

## Abstract

**Background:**

We present a technique for aortic composite graft implantation after left ventricular outflow tract destruction due to its proximal dehiscence.

**Case presentation:**

A 53-year-old gentleman with rheumatoid arthritis and history of Bentall procedure, presented with heart failure symptoms for the past month. Transthoracic echocardiogram identified prosthetic valve dysfunction, and transesophageal echocardiogram detected that its mechanism was by dehiscence. After excluding infectious etiology, it was hypothesized that the cause was the absence of endothelialization, owing to immunosuppressive therapy. Repair surgery was successful, and 2 years later, the patient is fully asymptomatic.

**Conclusions:**

Immunosuppressive drugs are a rare cause of aortic composite graft dehiscence. Left ventricular outflow tract surgical reconstruction remains an extremely complex and high-risk intervention, with the need for reentry into cardiopulmonary bypass and graft proximal segment implantation in a lower position.

## Background

Aortic composite graft dehiscence is an unusual complication, reported in 0.1–1.3% of the patients [1]. The most common predisposing factor is infectious endocarditis, with only 3% of patients presenting with other causes, such as nonbacterial thrombotic endocarditis in 0.9–1.6% [2]. Immunosuppressive drugs may inhibit endothelialization, leading to dehiscence.

Bentall redo is an extremely complex procedure, especially because there is need for reentry into cardiopulmonary bypass and the left ventricular outflow tract is absent.

We present a technique for aortic composite graft implantation after left ventricular outflow tract destruction due to its proximal dehiscence, possibly caused by a rare cause of dehiscence, namely absence of endothelialization, due to immunosuppressive drugs.

## Case presentation

A 53-year-old male patient with a history of rheumatoid arthritis undergoing treatment with methotrexate, and a Bentall procedure with mechanical prosthesis (St Jude Medical™ nº28/25) implantation 2 years before, without complications. One year after the procedure, due to a complete atrioventricular block, a pacemaker was implanted. A transthoracic echocardiogram, dated 2 months before the present event, while asymptomatic, revealed moderate regurgitation of the aortic mechanical prosthesis.

**Fig. 1:**
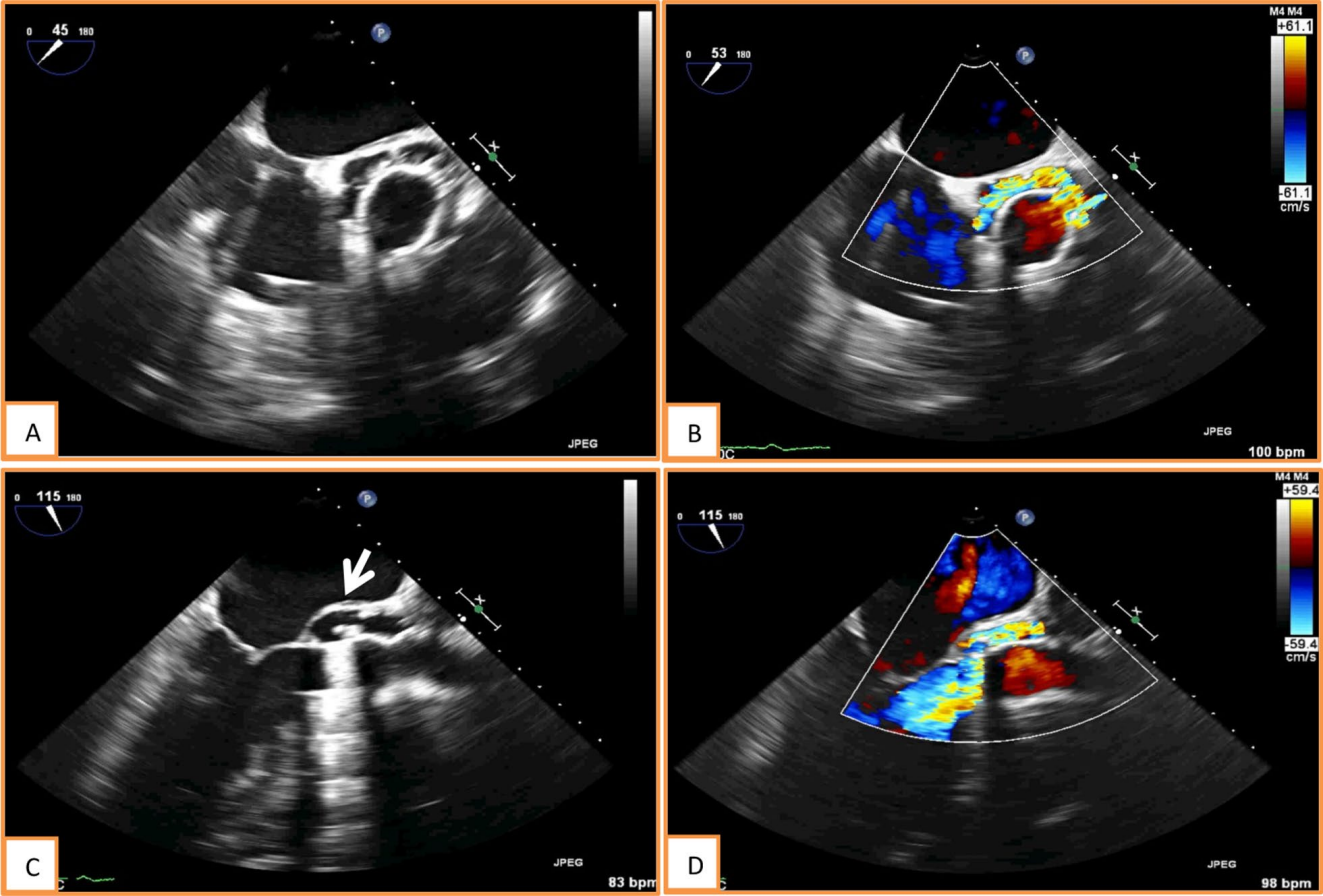
Transesophageal echo **A** short-axis (SA) view showing dehiscence (arrow); **B** SA color Doppler; **C** pseudoaneurysm in 3-chamber view (arrow); **D** 3-chamber color Doppler

The patient presented to our hospital with fatigue and breathlessness for low-intensity exertion since the previous month. He did not complain of fever or any infectious symptoms. Laboratory analysis revealed minor inflammatory parameters elevation. Transthoracic echocardiogram showed a dilated LV, ejection fraction of 49%, diffuse hypokinesis and severe prosthetic valve regurgitation. Transesophageal echocardiogram showed blood flow surrounding the prosthetic ring due to dehiscence, with rocking and severe regurgitation. There was also a fistula from the aortic root pseudoaneurysm to the LV (Fig. 1).

Angio-CT, performed to better characterize the complication, confirmed the presence of a large peri-prosthetic leak, on the left and posterolateral sides of the prosthesis.

It was decided to perform a second Bentall procedure. Surgery was carried out through a secondary median sternotomy, and adhesions lysis was performed. Cardiopulmonary bypass was established through the left femoral artery and the right atrial appendix. The patient was cooled to 30 °C, the distal ascending aorta was cross-clamped, and anterograde cardioplegia with a single dose of Custodiol® was administrated into coronary ostia. Then, the aortic conduit was transected.

**Fig. 2:**
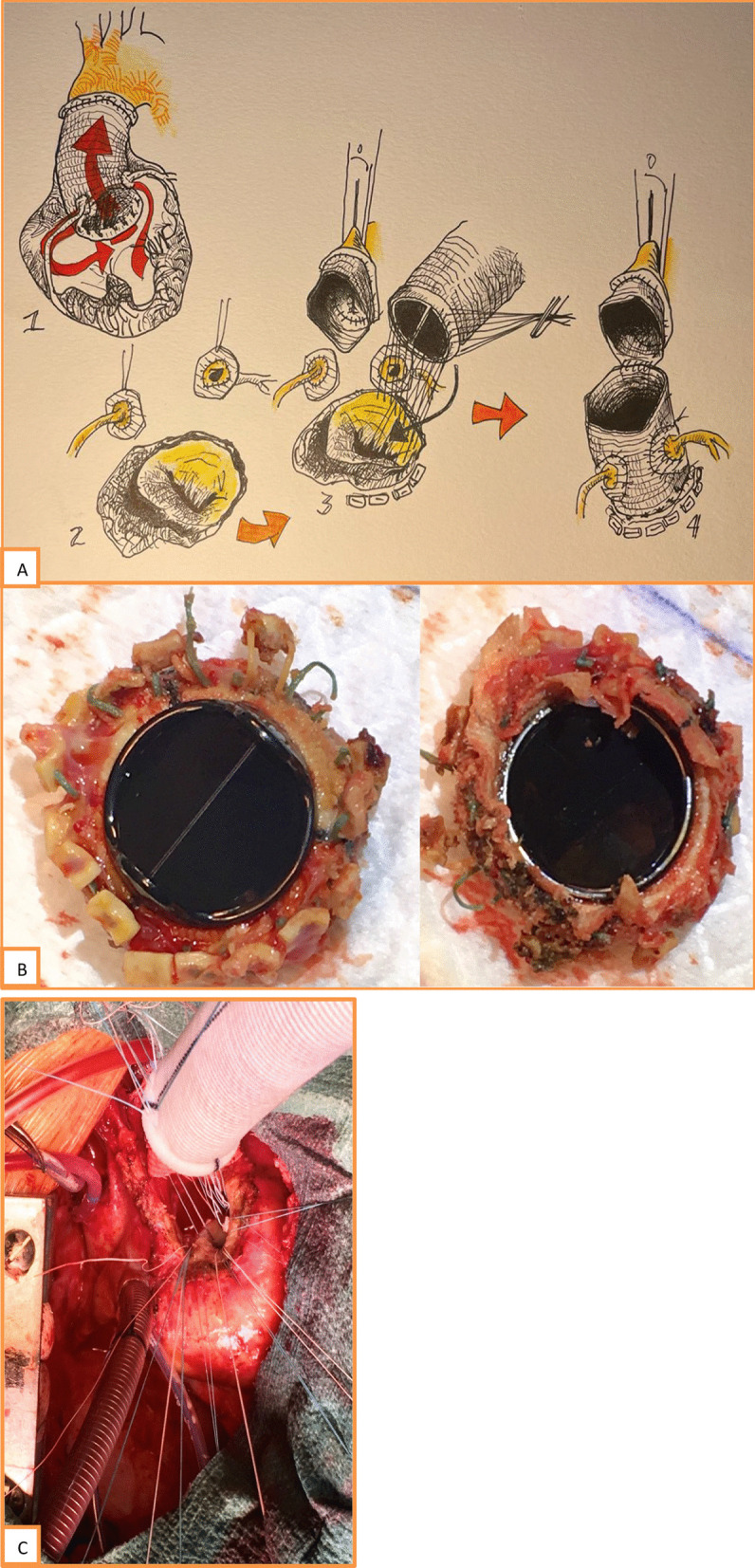
Surgical findings and technique **A** 1—Bentall proximal anastomosis dehiscent, postoperative adherences to surrounding tissues, and “rocking valve,” 2—pseudoaneurysm in aortoventricular discontinuity. Coronary buttons attached to a fringe of previous graft, 3—proximal anastomosis deep in LVOT, 4—coronary buttons implanted. Distal composite graft anastomosis to an original graft fringe; **B** unendothelialized aortic mechanical prosthetic valve; and **C** proximal suture

Intraoperative findings after conduit transection were compatible with almost complete dehiscence of the proximal anastomosis of the composite graft, which was kept in place due to coronary anastomosis and adhesions between the conduit and nearby structures. As the conduit was detached, there was blood flow between it and the adhesions, similar to an aortic root pseudoaneurysm. The usual prosthetic skirt endothelialization was not present (Fig. 2). Intraoperative findings were not compatible with present or past infection. No thrombus was found. Nonetheless, samples were sent to microbiology.

A new mechanical composite aortic valve graft (St Jude Medical™ nº 24/23) was implanted. Pledgeted U-sutures were placed circumferentially on the base of the anterior mitral leaflet, and on the interventricular septum, where the tissue is fibrous and resistant. By inserting the proximal portion of the composite graft on a lower level, it was possible to exclude the surrounding tissues, and debris of the first intervention. The composite graft was lowered into the left ventricular outflow tract, with the valve positioned under the aortic-mitral curtain level. The segments of the first conduit where the coronary ostia were initially sutured were reimplanted to the new one. The distal part of the new conduit was then sewed to the distal portion of the first one.

During hospital admission, all (pre- and postsurgical) blood cultures consecutively collected were negative. Series of infectious serologies were also negative. There was never evidence of infection, based on clinical signs, evolution of inflammatory parameters, serology, intraoperative findings and negative microbiology and pathology evaluations for infectious agents. However, it was decided to perform antibiotics after surgery with gentamycin (for 2 weeks) and vancomycin (for 6 weeks), as endocarditis is a major cause of prosthesis dehiscence.

There was never evidence of autoimmune disease recrudescence, based on clinical evaluation and serology.

Transesophageal echocardiogram reevaluations (during antibiotic course and 2 weeks after its conclusion) did not reveal any new findings.

As infectious or autoimmune endocarditis were not probable, it was hypothesized that the absence of prosthetic endothelialization was due to the immunosuppressive therapy, so the case was discussed with Rheumatology. It was decided to suspend methotrexate for more than 6 months, in order not to suppress valve endothelialization.

Two years later, the patient remains fully asymptomatic without any signs of heart failure. Transthoracic echocardiogram revealed complete recovery of LV dimension and global function, and absence of prosthetic valve dysfunction.

## Conclusions

First, in retrospect, the patient’s history of complete atrioventricular block may have been a consequence of the complication that the authors describe.

Furthermore, a second Bentall procedure, with the need for reentry into cardiopulmonary bypass, remains a very challenging and high-risk operation.

Third, the appropriate strategy to repair the LVOT remains a matter of debate. In this case, the proximal segment of the composite graft was implanted in a lower position and sutured to the LVOT and the base of the anterior mitral leaflet. Even though there was never evidence of infection, the above-described technique was performed to exclude an eventually infected area.

Finally, the authors assumed that dehiscence was a consequence of prosthesis unendothelialization, and not due to infectious or nonbacterial thrombotic endocarditis, as there was never clinical, analytic, macroscopic, microbiological or anatomopathological evidence of neither of these. Consequently, this was considered to be possibly a consequence of the patient’s immunosuppressed state, due to therapy with methotrexate [3]. It was decided to suspend methotrexate, for the endothelialization process to occur, and this was only restarted more than 6 months after the procedure, ensuring that healing was complete [4, 5].

This case highlights the LVOT surgical repair after an unusual cause of dehiscence—prosthetic < valve unendothelialization—due to immunosuppressive therapy. This diagnosis was assumed only after the exclusion of other causes, such as infective endocarditis and nonbacterial thrombotic endocarditis.

## Data Availability

Not applicable.

## Abbreviations

LV: Left ventricle
LVOT: Left ventricular outflow tract
